# Acute and Subacute Effects of Session with the EXOPULSE Mollii Suit in a Multiple Sclerosis Patient: A Case Report

**DOI:** 10.3390/bioengineering12090994

**Published:** 2025-09-18

**Authors:** Serena Filoni, Francesco Romano, Daniela Cardone, Roberta Palmieri, Alessandro Forte, Angelo Di Iorio, Rocco Salvatore Calabrò, Raffaello Pellegrino, Chiara Palmieri, Emanuele Francesco Russo, David Perpetuini, Arcangelo Merla

**Affiliations:** 1 Unit of Neuro-Rehabilitation, IRCCS “Casa Sollievo della Sofferenza”, 71013 San Giovanni Rotondo, Italy; 2 Department of Engineering and Geology, University “Gabriele D’Annunzio” of Chieti-Pescara, 65127 Pescara, Italy; 3 Child and Adolescent Neuropsychiatry Unit, Department of Mental Health, ASL Foggia, 71121 Foggia, Italy; 4 Health Rehabilitation Management (HRM SRL), 71013 San Giovanni Rotondo, Italy; 5 Department of Innovative Technologies in Medicine & Dentistry, University “Gabriele d’Annunzio” of Chieti-Pescara, 66100 Chieti, Italy; 6 IRCCS Centro Neurolesi Bonino-Pulejo, S.S. 113 Via Palermo, C.da Casazza, 98124 Messina, Italy; 7 Department of Medicine and Surgery, LUM University, 70010 Casamassima, Italy; 8 Clinica Medica, Department of Internal Medicine, Marche University Hospital, 60126 Ancona, Italy; 9 Padre Pio Foundation and Rehabilitation Centers, 71013 San Giovanni Rotondo, Italy

**Keywords:** multiple sclerosis (MS), transcutaneous electrical stimulation (TENS), cerebral plasticity, electroencephalography (EEG), heart rate variability (HRV), infrared thermography (IRT)

## Abstract

Multiple sclerosis (MS) is a chronic neurological disease often resulting in motor and autonomic dysfunction. This case report investigates the acute and subacute effects of the EXOPULSE Mollii Suit (EMS), a wearable device capable of delivering transcutaneous electrical nerve stimulation to multiple anatomical regions, in a 43-year-old woman with MS. The patient underwent a clinical evaluation before the EMS treatment, during which central nervous system (CNS) and autonomic nervous system (ANS) responses were monitored using electroencephalography (EEG), heart rate variability (HRV), and infrared thermography (IRT). Immediately after the first EMS application, the clinical evaluation was repeated. The intervention continued at home for one month, followed by a post-treatment evaluation similar to the pre-intervention assessment. Functional evaluations showed improvements in sit-to-stand performance (from 8 s to 6 s), muscle tone (MAS scale for the right side from 3 to 2 and for the left side from 2 to 1), clonus, and spasticity (from 3 to 2). EEG results revealed decreased θ-band power (on average, from 0.394 to 0.253) and microstates’ reorganization. ANS activity modifications were highlighted by both HRV (e.g., RMSSD from 0.118 to 0.0837) and IRT metrics (e.g., nose tip temperature sample entropy from 0.090 to 0.239). This study provides the first integrated analysis of CNS and ANS responses to EMS in an MS patient, combining functional scales with multimodal instrumental measurements, emphasizing the possible advantages EMS for MS treatment. Although preliminary, these results demonstrated the potentiality of the EMS to deliver effective and personalized rehabilitative interventions for MS patients.

## 1. Introduction

Multiple sclerosis (MS) is a chronic autoimmune disease characterized by inflammation, demyelination, and neurodegeneration, which impact the central nervous system (CNS). This condition majorly affects young adults and can lead to several neurological symptoms, such as cognitive, sensory, and motor impairments [1]. In detail, the pathophysiology of MS involves the immune-mediated destruction of myelin, resulting in a progressive decline in motor function, which can significantly impact patients’ daily activities and quality of life [2,3]. Motor impairments in MS patients include muscle weakness, loss of muscle control, abnormal muscle tone, ataxia, and fatigue [1,4]. Notably, motor dysfunction also affects psychosocial aspects of patients’ lives. For instance, gait impairments can lead to increased risk of falls, social isolation, and decreased participation in community activities, all of which contribute to a lower quality of life [2,5]. From this perspective, improving motor function of MS patients is a crucial goal of the therapeutic treatments used in clinical practice. To this aim, current therapies aimed at enhancing motor functions in MS patients encompass pharmacological treatments, physical rehabilitation, and neuromodulation techniques. Pharmacological therapies primarily aim to reduce the frequency of relapses and slow disease progression. Importantly, these medications do not directly enhance motor function, but they can help preserve neurological function over time, indirectly enhancing motor capabilities [6]. Physical rehabilitation comprises exercise programs tailored to individual capabilities to improve muscle strength, balance, and overall physical fitness [7,8]. Neuromodulation techniques, including transcranial magnetic stimulation (TMS) and vagus nerve stimulation (VNS), are emerging as promising tools for motor recovery. TMS has been utilized to enhance cortical excitability and facilitate motor learning [9], whereas VNS has shown potential in promoting neuroplasticity, which may help in motor function recovery after demyelination [10]. Among the neuromodulation techniques, transcutaneous electrical nerve stimulation (TENS) is a non-invasive technique that delivers low-voltage electrical currents through the skin to stimulate peripheral nerves, which can modulate pain perception and promote neuroplastic changes in the CNS [11]. Notably, the effectiveness of TENS to enhance outcomes across the International Classification of Functioning (ICF) domains [12] of body structure and function (e.g., clinical Modified Ashworth Scale, MAS) and activity (e.g., walking and moving) has been underscored by several recent systematic reviews [13,14,15]. Regarding the TENS application for MS treatment, studies have shown that TENS can effectively reduce spasticity, improve motor function, and enhance patients’ quality of life [16]. Despite the promising findings, the efficacy of TENS in MS treatment remains a topic of debate. In fact, some studies demonstrated that while TENS may be beneficial for certain symptoms, its effectiveness can vary among individuals [16]. This variability calls for further research to establish standardized protocols and identify which patient populations may benefit most from TENS therapy.

The Mollii Suit (Exoneural Network AB, Danderyd, Sweden) is a full-body garment equipped with 58 electrodes, which deliver low-frequency electrical stimulation to selected muscle groups. It is administered to treat spasticity, motor impairments, and chronic pain of neurological origin associated with pathologies such as cerebral palsy, multiple sclerosis, stroke, and acquired brain or spinal cord injuries. The device aims to reduce spasticity and pain while supporting improvements in mobility, flexibility, and range of motion [17]. The incorporation of TENS into a wearable format has several benefits for rehabilitation objectives. The electrodes affixed to the suit’s body fabric enable accurate targeting of certain muscle groups. This enables the design of personalized treatment to address the individual’s unique needs. Furthermore, the wearable capabilities of EMS allow patients to participate in treatment while executing functional tasks and motions. The latter feature enables users to engage in activities of daily living and functional duties, hence increasing the likelihood of regular and frequent utilization, which is crucial for achieving optimum therapeutic results [18]. Importantly, it should be noted that studies investigating the CNS and autonomic nervous system (ANS) activities in response to the EMS treatment are lacking. Monitoring both the CNS and the ANS responses allows for the evaluation of the effects of the treatment on the neuroplasticity and the psychophysiological condition of the patient, reflecting the acceptance of the garment by the users. To this goal, a multimodal approach able to measure brain activity and peripheral biosignals influenced by the ANS is fundamental.

In order to evaluate the effects of the EMS on the CNS during and after the stimulation, it could be crucial to monitor brain activity through portable neuroimaging techniques. To this aim, electroencephalography (EEG) is a powerful tool for evaluating brain plasticity. EEG is a non-invasive, portable scalp-located neuroimaging technique able to measure the electrical activity of the brain through electrodes placed on the scalp. It is characterized by a high temporal resolution, which allows for the detection of rapid changes in brain activity and can be employed to explore the mechanisms of neuroplasticity. For instance, studies have demonstrated that specific EEG patterns, such as alpha (α) and gamma (γ) oscillations, are associated with different states of cortical excitability and can predict the outcomes of plasticity-inducing interventions [19,20]. Importantly, a novel approach for EEG data analysis increasingly recognized as a valuable tool for assessing brain plasticity relies on the evaluation of the EEG microstates, which are quasi-stable patterns of electrical activity in the brain that last approximately 60 to 120 milliseconds [21]. The EEG microstates aim to decompose the continuous EEG signal into a finite number of discrete microstates that reflect coherent neuronal activity [22]. Microstates are typically classified into four canonical classes, each associated with different functional networks in the brain. For instance, microstate A is associated with the salience network, while microstate B is related to the default mode network [23,24]. Hence, some features of the microstates, such as occurrence and duration, can provide insights into the underlying neural mechanisms of cognitive processes, including attention, memory, and emotional regulation [25,26]. Notably, alterations in the dynamics of these microstates can be indicative of several neurological and psychiatric conditions, such as schizophrenia, depression, and Alzheimer’s disease [24,27,28].

Investigating the physiological responses to the treatment is a crucial aspect to consider when assessing the effectiveness of the EMS. Importantly, TENS has been shown to influence the balance between sympathetic (SNS) and parasympathetic (PNS) nervous system activity [29] and to induce pain relief [30]. In addition, changes in autonomic responses can indicate how well patients are coping with the treatment, helping the clinicians in adjusting treatment parameters to enhance patient comfort and adherence [31]. To this aim, the employment of wearable and contactless technologies able to monitor physiological signals related to the ANS activity could offer information regarding the effectiveness and acceptance of the EMS-based treatment [32]. Among the variety of methods available to this goal, the heart rate variability (HRV) is a well-established parameter to assess the ANS activity, also providing information regarding the balance between SNS and PNS [33,34]. Information regarding the HRV is usually obtained through electrocardiography (ECG) and photoplethysmography [35], particularly when the sensors are embedded in wearable devices such as smartwatches, smart jewelry, and smart t-shirts [36].

Another relevant tool for ANS activity assessment is infrared thermography (IRT). This non-invasive imaging technique measures surface temperature, which is related to physiological blood flow modulations and autonomic regulation [37,38]. In detail, the ANS influences skin temperature through its control of blood vessel dilation and constriction. Therefore, changes in skin temperature can be suggestive of ANS activity, providing, in this context, information on the physiological responses to therapeutic interventions [37]. Notably, IRT can provide real-time feedback on the physiological effects of therapy, which can enhance patient engagement and acceptance of treatment [39]. Importantly, in the literature no studies combining EEG microstate analysis, HRV, and IRT in EMS-treated patients with MS are reported.

In this perspective, the aim of this study is to assess modifications in the ANS and CNS activity in response to a session with the EMS and after one month of treatment through HRV, IRT, and EEG on a patient affected by MS. Specifically, the patient exhibited reduced functional mobility and spasticity, thus clinical tests widely used in MS to provide meaningful information on lower-limb strength and altered muscle tone have been employed. Additionally, since MS is frequently associated with alterations in cortical excitability and network dynamics [40], the EEG was applied to monitor the brain activity.

Finally, considering the high prevalence of autonomic dysfunction in MS [41], HRV and IRT were used to provide a non-invasive measure of ANS activity.

## 2. Materials and Methods

### 2.1. Study Design and Participant

The present study consists of a case report investigating the acute and subacute effects of a treatment session with the EXOPULSE Mollii suit on a multiple sclerosis patient. The participant was a 43-year-old woman with secondary progressive multiple sclerosis, diagnosed in 2006. The patient began treatment with Ocrelizumab in September 2018 and completed the treatment in December 2023. Ocrelizumab is a humanized recombinant monoclonal antibody targeting CD20, which selectively binds to CD20-expressing B cells. CD20 is a surface antigen found on pre-B cells, mature B cells, and memory B cells, but it is not expressed on lymphoid stem cells or plasma cells. The mechanism of action involves immunomodulation through the reduction in the number and function of CD20-positive B cells.

The initial dose, approximately two years prior, was 600 mg, administered as two separate intravenous infusions: a first infusion of 300 mg followed by a second infusion of 300 mg two weeks later. Subsequent doses were administered as a single 600 mg intravenous infusion every six months. The treatment was monitored every six months through neurological evaluations, follow-up MRI scans, and periodic blood tests. The patient did not report or experience any adverse events or side effects.

In addition, the patient began treatment with amantadine 100 mg in 2014. Amantadine is an antiviral used in the prevention and early treatment of influenza A virus infections; it also improves mild disabilities caused by bradykinesia, as well as tremor and rigidity in Parkinson’s disease. The patient is currently still undergoing treatment with amantadine.

During the neuro-suit stimulation protocol, the patient was followed up with close neurological monitoring and underwent regular brain and spinal cord MRI scans with and without contrast agent, blood tests, neurological assessments, and evaluation scales. The disease had been declared stable for approximately two years at the time of enrollment, and her EDSS score had remained at 7 for about two years. Furthermore, the patient receives intravesical botulinum toxin injections for neurogenic bladder management. She is alert, conscious, oriented, and cooperative. Postural changes and transfers are possible with adaptations. Standing is feasible with support; ambulation is possible with moderate assistance and a cane for very short distances (10 m). For longer distances, the patient uses a wheelchair. The patient exhibited a paraparetic/ataxic gait with a cautious, small-stepped pattern. The Romberg test was positive. During the finger-to-nose test, the patient exhibits a tendency toward retropulsion. Bilateral single-leg stance is not achievable. Coordination assessment reveals left-sided paresis in the upper limbs, accompanied by an impaired pincer grasp. Muscle tone is increased in the left pectoralis major and biceps brachii, while muscle trophism is reduced but remains within normal limits. A mild weakness is also observed on the right side. Deep tendon reflexes are hyperreflexive, and sensory examination indicates distal hypoesthesia and paresthesia.

In the lower limbs, there is severe left-sided paresis with distal plegia affecting the tibialis anterior, extensor digitorum longus, and gastrocnemius–soleus complex, with a Medical Research Council (MRC) muscle grade of 1/5. Right-sided paresis is also present. Spastic hypertonia is noted in the rectus femoris and biceps femoris, with positive Duncan–Ely test and Tardieu signs, as well as in the triceps surae, evidenced by a positive Silverlskiold test bilaterally. Inexhaustible clonus and marked muscle atrophy are observed. Deep tendon reflexes are hyperreflexive, particularly in the patellar and Achilles reflexes. The right ankle is edematous, with increased thermotactile sensitivity and a measured circumference difference of +1 cm.

Additional findings include the absence of dysphagia; however, there is a delayed onset of the swallowing reflex following COVID-19 infection. The patient has a neurogenic bladder requiring self-catheterization and a neurogenic bowel managed with Movicol therapy. There have been no recent falls. While there is no dyspnea, the patient reports experiencing easy fatigability.

The participant signed a written consent form, and she could withdraw from the experiment at any time. The current study was conducted in accordance with the ethical standards recognized by the Declaration of Helsinki.

### 2.2. Intervention

**Figure 1:**
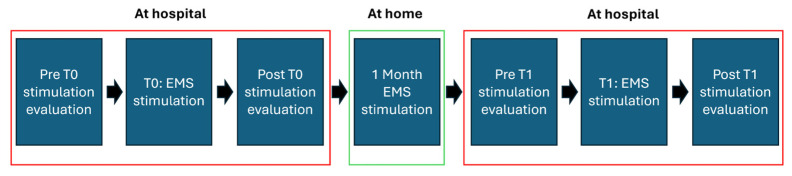
Timeline of the study. In the red boxes are grouped the phases performed at hospital, whereas the green box reports the phase carried out at home.

The experiment was composed of two separate sessions 1 month apart. During the first session, the motor function evaluation was performed both before and after the stimulation, whereas in the second session the evaluation was performed only after the EMS treatment. Notably, the motor function evaluation was performed at the same time of day (at 11 a.m.) for both sessions. In the month between the first and second sessions, the patient underwent EMS sessions three times per week, for a total of 12 EMS sessions. The stimulation lasted 60 min, activating all 58 electrodes and using a stimulation pattern tailored for the peculiar patient. During the stimulation, the patient was positioned supine on a bed. This study design allowed for the evaluation of the acute effects of the stimulation during the first session and sub-acute effects during the second session. The timeline of the study is described in Figure 1.

### 2.3. EMS Configuration

The suit was provided to the patient following a physiatric assessment, during which indications and contraindications were evaluated. It was granted through a free loan-for-use agreement. The free rental period took place between July and September 2022. The suit was provided by the Reggio Emilia branch of Otto Bock Soluzioni Ortopediche SRL US (Reggio Emilia, Italy). The serial numbers were as follows:

- Jacket, size W/XL—model and serial number: 100-1-12575
- Trousers, size W/XL—model and serial number: 100-1-12604
- Control unit: 100-4-1331

The software used to program the suit was “Molliisoft”, version 1.2. The stimulation pattern was tailored based on the patient’s spasticity profile. The parameters of the stimulation were fixed based on initial response and tolerance and monitored to ensure safety, therapeutic efficacy, consistency during the one-month treatment period, and avoid inadvertent changes by the patient. Notably, the EMS does not disclose a formal open-source algorithm to tailor the treatment to the patients, but its clinical use is guided by a semi-standardized protocol based on initial assessment, initial parameter setting, customization based on response, and locking parameters.

The electrical stimulation parameters were carefully individualized by the clinical team at T0 based on the patient’s characteristics: diagnosis, symptom severity, age, and muscle mass of the targeted muscle group undergoing functional stimulation.

The stimulation is set at 20 V, 20 Hz, while the pulse widths, expressed in microseconds, vary. In the presented scheme, these vary from 25 (point 1) to 175 (point 30) microseconds.

**Figure 2:**
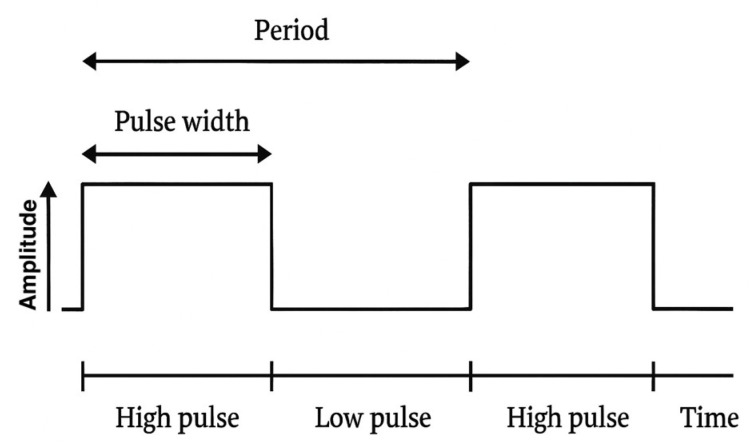
Waveform used in the stimulation protocol and its characteristics.

Each point increases the stimulation by 5 microseconds (point 2 = 30 microseconds; point 3 = 35 microseconds, and so on). The waveform used in the stimulation protocol is reported in Figure 2.

The stimulation applied to the patient aims to improve foot pronation–supination through significant activation of the tibialis anterior muscles, considering the muscular tension (95 microseconds on the right, 110 microseconds on the left). Similarly, hyperextension of the knee was addressed through stimulation of the muscle group responsible for knee flexion (65 microseconds on the right, 75 microseconds on the left), and hip flexion was facilitated by stimulating the hip flexors (65 microseconds bilaterally). Additionally, targeted stimulation was applied to the hip abductors (90 microseconds on the right and 105 microseconds on the left). All these stimulations aim to improve gait pattern, reduce extensor synergy, and strengthen balance in both static and dynamic conditions.

Regarding the upper limbs, due to reduced endurance and hand functionality, with greater impairment on the left side, stimulation was introduced for the scapular girdle (35 microseconds in all segments), the triceps to support elbow control (50 microseconds on the right, 60 microseconds on the left), and the muscles responsible for wrist flexion (65 microseconds on the right and 80 microseconds on the left).

According to the stimulation protocol for this diagnosis, stimulation was applied to stabilize posture at the level of the abdominal muscles (50 microseconds, bilaterally) and the paravertebral muscles (60 microseconds, bilaterally).

**Figure 3:**
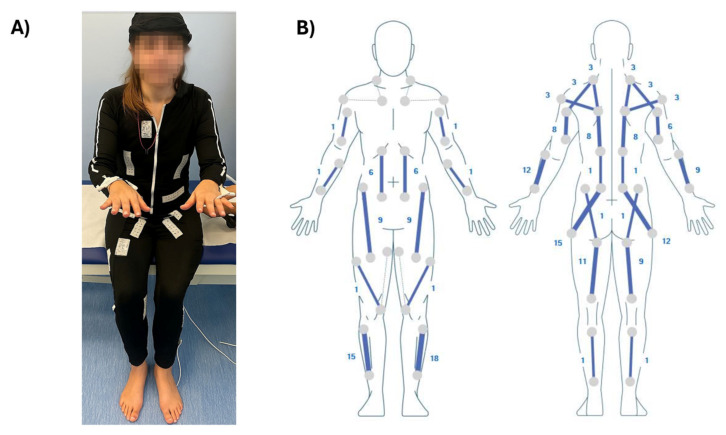
(**A**) The EMS worn by the participant and (**B**) the graphical scheme of the electrical stimulation used for the intervention. The numbers indicate the probes for electrical stimulation.

Finally, a light multi-site stimulation (25 microseconds) was applied to all segments not involved in functional support stimulation, following the general stimulation protocol with the EMS, to enhance the overall relaxation effect. The EMS worn by the participant and the graphical scheme of the electrical stimulation delivered by the device are reported in Figure 3A and Figure 3B, respectively. The selected muscle groups and stimulation settings (pulse width) employed in the EMS for the intervention are reported in Table 1.

### 2.4. Clinical Scales

The following clinical scales were evaluated for the patient. Notably, all variables were evaluated in basal conditions (i.e., prior to the intervention) and after the intervention.

- Sit to Stand (STS): it assesses lower limb strength, balance, and endurance by measuring the time or number of repetitions a person takes to move from a sitting to a standing position.
- Timed Up and Go (TUG): it is a test used to assess mobility, balance, walking ability, and fall risk. During the test, the participant starts seated, stands up, walks a short distance (typically 3 m), turns, returns, and sits down again while the time is recorded.
- Clonus Test: it is a neurological examination used to assess involuntary, rhythmic, and repetitive muscle contractions, typically in the lower limbs, to evaluate upper motor neuron lesions.
- Pendulum Test: it is used to evaluate spasticity by observing the passive swinging motion of the lower limb after an initial impulse.
- VAS (Visual Analog Scale): it is a subjective measure of pain intensity, usually represented as a 10 cm line where one end represents “no pain” and the other represents “worst pain imaginable.” The patient has to mark a point on the line to indicate their perceived pain level.
- Modified Ashworth Scale (MAS) Tone Test: it is a clinical tool used to assess muscle spasticity by measuring resistance during passive soft-tissue stretching.
- Penn Spasm Frequency Scale: it is a scale used to assess the frequency and severity of muscle spasms in individuals with neurological conditions, particularly spinal cord injuries.
- Modified Fatigue Impact Test Scales (MFIS): it is a questionnaire-based assessment that measures the impact of fatigue on physical, cognitive, and psychosocial functioning in individuals with neurological disorders, particularly MS.

### 2.5. Instrumental Measures

Instrumental measures were taken during the motor function evaluation as well as during the stimulation during both the first and second session. The EEG data were recorded through a 9-channels device (Encephalan Mini AP-10 system). The impedance between scalp and electrodes was checked before each recording in order to collect signals with good quality. The sample frequency was 250 Hz. The electrodes were placed in accordance with the 10–20 standard, covering the whole head.

The same device was used to measure the 1-lead ECG, with a sample frequency of 250 Hz.

The IRT signals were acquired through FLIR SC660 thermal infrared camera (Teledyne FLIR, Wilsonville, OR, USA). It is characterized by a 640 × 480 bolometer FPA, a sensitivity/noise equivalent temperature difference of 30 mK at 30 °C, and a field of view of 24° × 18°. The camera was positioned 60 cm from the subject and the sampling rate was set to 10 Hz. Importantly, the camera was calibrated using a blackbody to reduce optical artefacts and any sensor response drift or shift. To mitigate thermoregulatory influences, IRT measurements were performed in compliance with thermal measurement standards [42]. In particular, the recordings were carried out in a thermoneutral environment to minimize thermoregulatory-induced alterations. Additionally, the patient was given a 15-min acclimation period prior to the session to allow thermal stabilization with the surrounding environment [43]. Notably, to minimize potential influences of circadian rhythm, all sessions were conducted at a consistent time of day [44].

### 2.6. Data Analysis

Regarding the EEG data, a band-pass filter with cut-off frequencies of 1 Hz and 80 Hz, in conjunction with a notch filter at 50 Hz, was used (zero-lag 2nd order Butterworth digital filters). EEG epochs that were saturated or distorted were discarded by visual assessment. Additionally, cardiac and ocular artifacts, together with muscular activity contaminations, were eliminated using a semiautomatic technique grounded on independent component analysis (ICA) [45]. Specifically, blind source separation is mainly performed through ICA, since brain and artifactual independent components (ICs) exhibit recognizable patterns. Relying on deep-learning ability of self-extracting the features of interest, a CNN for off-line, automatic artifact identification through ICs developed in [45] was employed.

The pre-processed EEG data were analyzed over five pertinent frequency bands (θ-band: 3.5–8.2 Hz, α-band: 7.4–13 Hz, β-band: 13–30 Hz, δ-band: 1–4 Hz, γ-band: 26–40 Hz), and the power temporal envelopes were evaluated as the absolute values of their Hilbert transform.

In addition, the microstates segmentation was performed. The global field power (GFP) was computed as reported in Equation (1) [46]:

(1) $${GFP}_{t}=\sqrt{\frac{\sum_{i=1}^{n}u_{i}^{2}}{n}}$$

where *n* is the number of channels and *u* is the amplitude in µV at time *t*.

The time points corresponding to local maxima of GFP were identified, and the most representative scalp topographies were extracted using a k-means clustering algorithm. The clustering procedure was performed across a range of 2 to 20 clusters, and the optimal number of clusters was determined using the Krzanowski–Lai criterion [47]. This analysis revealed that the optimal number of microstates was k = 4. Subsequently, the patient’s EEG data were segmented according to these four prototypical microstate maps by applying a temporal smoothing procedure with a 12-millisecond window size [48]. As a result, a time series vector was generated, labeling each time point with one of the four identified microstates.

To characterize each of the four microstates, the following metrics were computed [49]:

- Mean Duration (Mean_Dur)
- GFP
- Temporal Coverage
- Occurrence Rate
- Hurst Exponent
- Global Explained Variance (GEV)

The ECG data processing included band-pass filtering the signals with cut-off frequencies ranging from 1 to 40 Hz. The R-peaks were detected, and the RR interval was assessed, enabling the analysis of both time- and frequency-domain measures. The following metrics have been calculated in detail:

- The Standard Deviation of Normal Heartbeat Intervals (SDNN): it quantifies the overall variability in heart rate by measuring the standard deviation of the intervals between successive normal heartbeats (NN intervals). It indicates the activation of both the sympathetic and parasympathetic nervous systems.
- The Root Mean Square of Successive Differences (RMSSD): it assesses short-term changes in heart rate.
- The Low-Frequency Power (LF, 0.04–0.15 Hz): it signifies a combination of sympathetic and parasympathetic activity, while it is often linked to sympathetic dominance.
- The High-Frequency Power (HF, 0.15–0.40 Hz): it indicates parasympathetic (vagal) activity and correlates with relaxation, deep respiration, and recuperation.
- The Low-Frequency to High-Frequency Ratio (LF/HF): it serves as a measure of autonomic equilibrium, with elevated values signifying heightened sympathetic dominance and diminished values reflecting enhanced parasympathetic activity.

For the analysis of the infrared thermography (IRT) signals, the quality of the thermal recordings was initially verified through visual inspection, and no recordings were excluded. Six regions of interest (ROIs) were defined: the nose tip, left and right nostrils, chin, perioral region, and corrugator area. The localization of these ROIs across video frames was achieved by tracking the nose tip using a dedicated algorithm [50]. In instances where the tracking algorithm failed—typically due to substantial head movement—the affected segments were corrected by interpolating values based on the mean of six samples preceding and following the motion artifact. This tracking procedure allowed for the extraction of the temporal dynamics of temperature within the selected ROIs. The following metrics were evaluated for each ROI:

- Mean Temperature (T_avg): Represents the average thermal value over time, indicating baseline skin temperature.
- Standard Deviation (T_std): Measures the variability of the temperature signal, reflecting fluctuations in thermal activity.
- Kurtosis (T_kurt): Quantifies the peakedness of the temperature distribution, highlighting the presence of extreme values.
- Skewness (T_skew): Assesses the asymmetry of the temperature distribution, indicating directional bias in thermal shifts.
- Sample Entropy (T_SampEn): Evaluates the complexity and regularity of the thermal signal, with lower values suggesting more predictable patterns.
- Low-Frequency Power (T_LF): Captures slow oscillatory components in the thermal signal, often associated with sympathetic activity.
- High-Frequency Power (T_HF): Reflects faster fluctuations in the signal, generally linked to parasympathetic modulation.
- LF/HF Ratio (T_LF/HF): Indicates the balance between sympathetic and parasympathetic influences on thermal regulation.

## 3. Results

### 3.1. Clinical Scales Results

**Figure 4:**
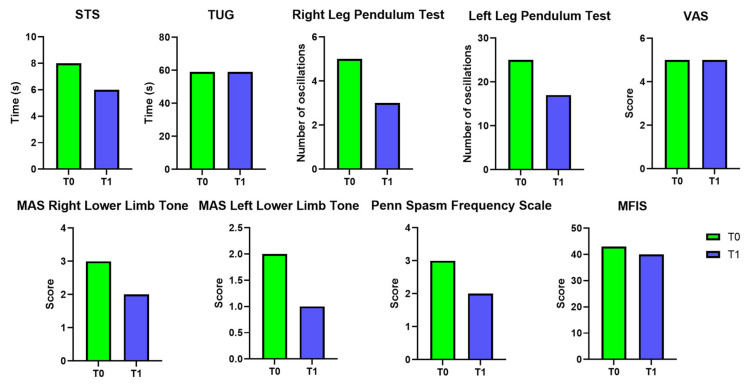
Clinical scales evaluated for the patient at T0 and T1.

Between T0 and T1, the STS test improved from 8 to 6 s, while the TUG test remained unchanged at 59 s. Clonus in the right lower limb changed from inexhaustible to absent, and in the left lower limb from inexhaustible to exhaustible. The Right Leg Pendulum Test decreased from 5 to 3 oscillations, and the Left Leg Pendulum Test from 25 to 17. Pain levels, as measured by the VAS, remained stable at 5. The Modified Ashworth Scale scores decreased from 3 to 2 for the right lower limb and from 2 to 1 for the left lower limb. The Penn Spasm Frequency Scale was reduced from 3 to 2, and the Modified Fatigue Impact Scale slightly decreased from 43 to 40. The results are reported in Table 2 and summarized in Figure 4.

### 3.2. EEG Results

**Figure 5:**
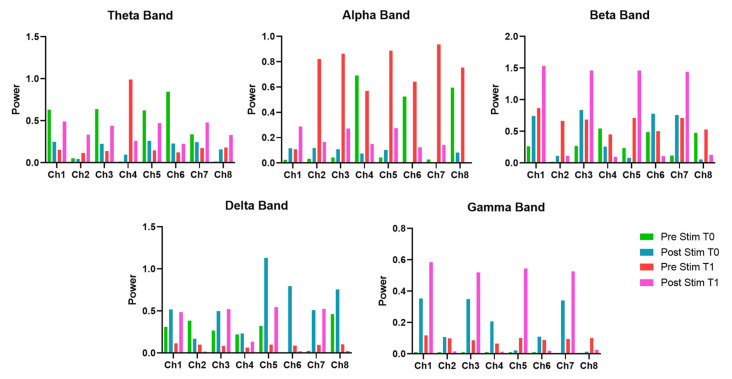
EEG power band for each channel evaluated for the patient at T0 and T1, before and after the EMS session.

Between T0 and T1, θ-band activity generally decreased during the pre-stimulus phase across all channels, with post-stimulus values at T1 showing an increase compared to pre-stimulus values. Regarding the α-band activity, it showed higher values in the pre-stimulus phase at T1 compared to T0, with a subsequent reduction in the post-stimulus phase across most channels. Concerning the β-band activity, it increased in the pre-stimulus phase at T1 in nearly all channels and remained elevated in the post-stimulus phase, particularly in channels 1, 3, 5, and 7. δ-band activity exhibited a general reduction in pre-stimulus values at T1 compared to T0, while post-stimulus values were relatively elevated, especially in channels 1, 3, 5, and 7. γ-band activity increased from T0 to T1 in both pre- and post-stimulus phases, with the most marked changes observed in post-stimulus values across several channels. The values of the EEG power bands are reported in Table 3 and summarized in Figure 5.

**Figure 6:**
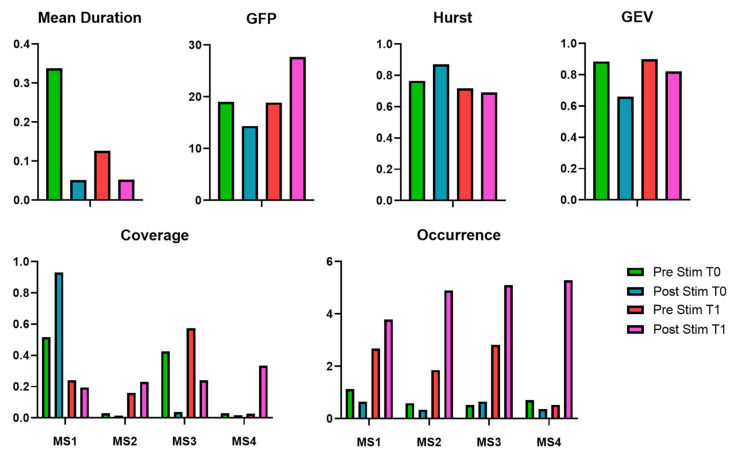
EEG microstates metrics evaluated for the patient at T0 and T1, before and after the EMS session.

Between T0 and T1, Mean_Dur decreased in the pre-stimulus phase and remained low in the post-stimulus phase. GFP remained stable in the pre-stimulus phase but showed a marked increase post-stimulus at T1. The Hurst exponent slightly decreased from T0 to T1 in both phases. GEV increased in the pre-stimulus phase from T0 to T1 and also improved in the post-stimulus phase. Regarding microstate coverage, state 1 showed a reduction from pre- to post-stimulus at T1, while states 2, 3, and especially 4 showed increased coverage in the post-stimulus phase. Occurrence rates for all four microstates increased substantially from T0 to T1, particularly in the post-stimulus phase, with the most pronounced rise seen in states 2, 3, and 4. The values and the percental changes of the evaluated EEG microstates are reported in Table 4 and summarized in Figure 6.

### 3.3. ECG Results

**Figure 7:**
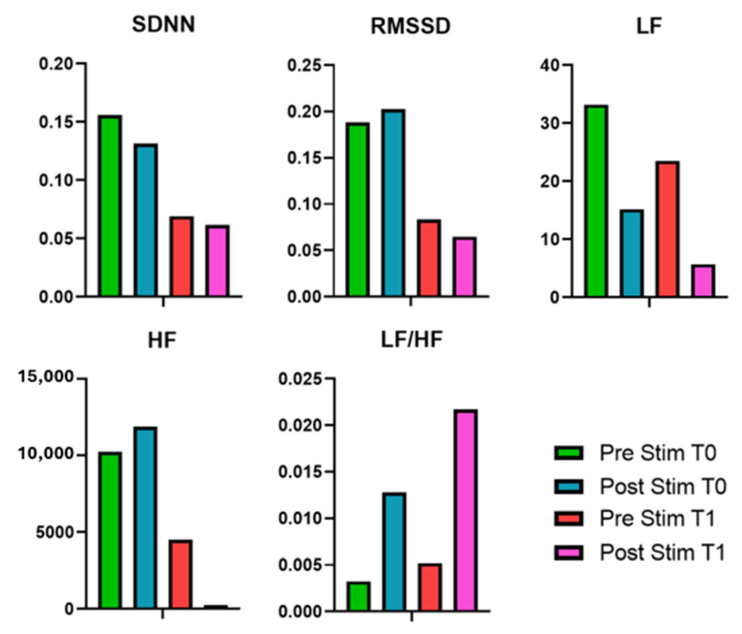
HRV metrics evaluated for the patient at T0 and T1, before and after the EMS session.

From T0 to T1, both SDNN and RMSSD decreased in the pre- and post-stimulus phases, indicating a general reduction in heart rate variability. LF power showed a substantial decline across all conditions, while HF power, initially high at T0, also decreased markedly at T1, especially in the post-stimulus phase. The LF/HF ratio decreased from T0 to T1 during the pre-stimulus phase and remained low in the post-stimulus phase, suggesting a shift toward parasympathetic dominance. The values and the percental changes of the evaluated EEG metrics are reported in Table 5 and summarized in Figure 7.

### 3.4. IRT Results

**Figure 8:**
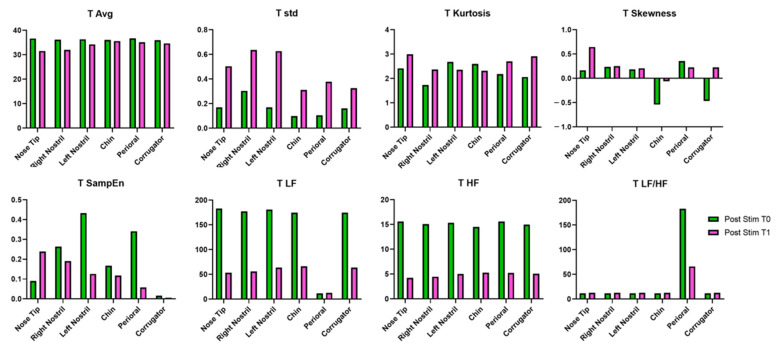
IRT metrics evaluated for the patient post EMS session at T0 and T1.

From T0 to T1 in the post-stimulus phase, a general decrease in average temperature (T_avg) was observed across all ROIs, with the most pronounced reduction at the nose tip. Temperature variability (T_std) increased notably in all regions, particularly in the left nostril and perioral areas. Kurtosis (T_kurt) generally increased in most regions, while skewness (T_skew) showed variable changes, with marked increases at the nose tip and corrugator. Sample entropy (T_SampEn) showed inconsistent trends, increasing only at the nose tip and decreasing elsewhere. Both LF and HF power decreased substantially across all ROIs, while the LF/HF ratio remained relatively stable or showed slight increases in all regions. The IRT results are reported in Table 6 and summarized in Figure 8.

## 4. Discussion

### 4.1. Functional Improvements

The present study is a novel case report study on the use of the EMS suit in an MS patient procedure that has never been carried out in a scientific setting. The clinical scales assessed before (T0) and after (T1) one month of EMS in a MS patient indicate notable functional improvements. The STS time decreased from 8 s to 6 s, suggesting enhanced lower limb strength and mobility. However, it should be highlighted that the TUG test remained stable, indicating that overall mobility and gait speed did not improve significantly. Regarding the spasticity-related tests, positive outcomes were observed in response to the EMS treatment. Specifically, the Clonus Test evaluated on the right lower limb shifted from “inexhaustible” to “absent” in response to the intervention, while the left lower limb improved from “inexhaustible” to “exhaustible.” These changes indicate a reduction in involuntary muscle contractions and hyperreflexia, suggesting that the EMS may have contributed to neuromuscular relaxation. Similarly, the Pendulum Test showed a decrease in the oscillation count from 5 to 3 for the right side and from 4 to 3 for the left side, supporting the trend of improved muscle tone regulation. Although these findings suggest that the EMS may be effective in reducing spasticity and improving functional lower limb performance in MS patients, further research is warranted to determine the long-term sustainability of these effects and to explore whether combining EMS with physical therapy could lead to more comprehensive mobility improvements.

Regarding the fatigue perception, the MFIS scale went from 43 to 40, demonstrating an improvement in this aspect. It is worth noting that the MFIS scale consists of three subscales (physical subscale, cognitive subscale, and psychosocial subscale), and the subscale that predominantly improved was the physical fatigue subscale, highlighting the impact of TENS on muscle and thus fatigue. Importantly, TENS is usually administered in reduced muscle groups, whereas the EMS is able to stimulate more body districts simultaneously. Hence, the marked perception of reduction in physical fatigue (rather than the other two components, cognitive and psychosocial) could be related to the simultaneous stimulation of numerous muscle groups involved in the perception of muscle fatigue [51,52].

It should be noted that no minimal clinically important difference has been firmly established for these tests in the MS population, hence the improvement observed could be considered potentially relevant, but the validation of MS-specific minimal clinically important difference thresholds is necessary.

### 4.2. CNS-Related Changes

The EEG band analysis before and after the EMS treatment provides insights into its impact on neural activity. Regarding the θ-band power, which is often associated with cognitive processing, memory, and relaxation, it showed a non-uniform response across channels. Specifically, at T0, the post-stimulation measurement generally showed a decrease in θ-band power on most channels, suggesting an immediate reduction in slow-wave activity, which could indicate increased cortical activation, thus reflecting a shift toward a more alert neural state. Notably, at T1, pre-stimulation values were lower compared to T0, suggesting a long-term reduction in baseline θ-band power. However, post-stimulation values showed an increase, indicating that EMS may facilitate a neuroplastic response over time. These results suggest that short-term EMS application reduces θ-band activity, potentially promoting wakefulness and cognitive engagement, while long-term application might modulate the brain’s baseline state, making it more responsive to stimulation.

In addition, the EEG microstate analysis highlights modifications in brain dynamics over both short- and long-term applications. In the short term, immediately after stimulation at T0, the mean duration of microstates decreased from 0.3381 to 0.0516, suggesting a reduction in the stability of microstate sequences. This modification underscores an increased rate of state-switching, reflecting heightened neural reactivity or modifications in cognitive processing. At the same time, GFP reduction associated with the EMS stimulation suggests a short-term reduction in overall brain activation. However, the Hurst exponent’s increase suggests a shift toward more persistent and stable neural dynamics. It is worth highlighting that GEV decreased from 0.8855 to 0.6604, reflecting a reduced ability of microstate templates to explain overall brain activity, which could suggest increased neural complexity or a transient state of reorganization.

Overall, the results indicate that short-term EMS application leads to a disruption of microstate stability, increasing state-switching and reducing the predictability of brain activity. This effect likely represents acute neural plasticity and reorganization. Over time, however, brain activity appears to adapt to the repeated stimulation, as reflected in the increased GFP and a return to a more diverse microstate dynamic. The observed increase in GFP at T1 post-stimulation suggests that prolonged TENS application may enhance overall neural excitability, which could potentially support cognitive function improvements in MS patients. These findings highlight the capability of EMS to modify the CNS activation patterns, but further research is necessary to determine whether these neurophysiological changes correlate with functional cognitive improvements and whether optimizing stimulation protocols could improve the long-term duration of these benefits.

### 4.3. ANS-Related Changes

Regarding the HRV metrics, the application of EMS over one month led to notable changes in HRV features. In detail, at T0, post-stimulation measurements showed an increase in RMSSD and HF power, suggesting an acute enhancement of PNS activity. At T1, baseline HRV values declined, suggesting a possible decrease in vagal tone. LF power showed a substantial increase post-stimulation at T0, followed by a further increase at T1 pre-stimulation, and by a significant reduction in post-stimulation. Conversely, HF power initially increased slightly at T0 post-stimulation but then dropped at T1 post-stimulation. These results suggest that while EMS may initially enhance autonomic flexibility, prolonged application could lead to compensatory shifts or autonomic fatigue. Hence, further investigation is needed to clarify the long-term effects of TENS on ANS function in MS patients, particularly regarding potential benefits over extended periods of stimulation.

The IRT data analysis reveals notable changes in thermal distribution secondary to the EMS treatment. T_avg at the nose tip decreased between the post-stimulation measurements at T0 and T1. This drop in temperature suggests a potential alteration in blood flow regulation, possibly due to autonomic nervous system modulation. Since the nose tip is particularly sensitive to SNS dependent vasoconstriction, this reduction may reflect increased SNS activity or a shift in thermoregulatory balance over time.

Additionally, T_std increased from T0 to T1 on the Nose Tip, suggesting a greater thermal dispersion, which could imply an irregular or more dynamic vasomotor response after one month of EMS application. Similarly, T_kurt and T_skew increased after 1 month of treatment, indicating modifications of the temperature distribution over time.

These findings indicate that EMS may influence facial temperature patterns, possibly by affecting SNS control over microcirculatory dynamics. The immediate post-stimulation effects at T0 showed relatively stable temperature dispersion, whereas over time, at T1, temperature variability increased, along with changes in distribution characteristics. This could reflect an adaptation of the ANS to repeated stimulation, leading to altered vascular responses. Further investigation is necessary to determine whether these thermographic changes correlate with broader autonomic adjustments and whether they hold clinical significance for individuals with neurological conditions such as MS.

### 4.4. Neurophysiological Correlates of Functional Improvements

This case report suggests that EMS was associated with reduced spasticity, as indicated by the Clonus and Pendulum Tests. These functional effects align with reports that TENS can improve strength and gait performance in people with MS and related populations, including case series using direct-current neuromuscular stimulation [53]. However, it should be highlighted that the TENS literature in MS shows mixed results. In fact, Miller et al., 2007 found not significant effect regarding the capability of TENS to reduce spasticity in MS patients [54]. Notably, this evidence may help explain why TUG remained unchanged for the investigated patient.

At the CNS level, the EEG θ-band reductions and altered microstate dynamics are supported by studies demonstrating that TENS modulates sensorimotor cortical excitability and oscillatory brain activity [55], while microstate metrics like duration, GFP, and GEV have been linked to shifts in large-scale brain network dynamics and plasticity [28,56]. These CNS-related modifications can be associated with the assessed improvement in functional lower limb performance and reduction in perception of physical fatigue.

The ANS-related findings are consistent with evidence that TENS can modify autonomic activity [57,58]. Moreover, the HRV findings are consistent with the IRT results. In fact, facial thermographic responses reflect ANS vasoconstriction modulating facial blood flow and autonomic balance [59]. In fact, an increased sympathetic activity is shown by the HRV metrics at T1 with respect to T0. Accordingly, the increased thermal variability observed in IRT at T1 likely reflects a long-term adaptation of sympathetic vasomotor control. The nose tip, highly sensitive to sympathetic-mediated vasoconstriction [59], showed greater dispersion and irregularity of temperature distribution after repeated stimulation, suggesting a more dynamic vascular response. Moreover, the reduction of SampEn of the temperature time course has been associated with increased sympathetic activity [60].

Finally, these findings demonstrate that EMS influences cortical excitability and autonomic regulation, which may foster specific functional gains such as improved lower-limb strength and reduced spasticity.

### 4.5. Strengths and Limitations

This case report has several strengths. First, it adopts a multimodal strategy, combining functional scales (STS, TUG, spasticity tests, fatigue questionnaires) with neurophysiological measures (EEG, HRV, and IRT). This integrated approach provides a more comprehensive insight of how EMS may influence motor function, cortical dynamics, and autonomic regulation in MS. Second, the study applied a longitudinal design, evaluating both immediate (T0) and one-month (T1) responses, allowing for distinguishing between acute and adaptive effects of stimulation. Finally, the detailed reporting of stimulation parameters and outcome measures enhances reproducibility of the findings.

Nonetheless, important limitations must be acknowledged. Findings are based on a single patient, limiting generalizability across the heterogeneous MS population. Moreover, the absence of a control condition prevents us from assessing eventual placebo effects or natural symptom fluctuations. Furthermore, evaluator blinding was not feasible in this single-case design. This limitation is particularly relevant for subjective measures such as spasticity scales and fatigue questionnaires, which may be more susceptible to observer bias. Additionally, follow-up was restricted to one month, hence further studies enlarging the evaluation period should be performed. Finally, the results are protocol-specific, thus it should be investigated whether different EMS parameters would yield more effective outcomes. Importantly, it should be noted that these findings are hypothesis-generating, thus future studies including randomized controlled and crossover protocols with extended follow-up and evaluator blinding should be implemented.

## 5. Conclusions

This case report describes the functional and neurophysiological changes in a patient with MS induced by EMS treatment. The results demonstrate improvements in clinical scales related to strength, spasticity, and motor performance and reduction of perceived physical fatigue, accompanied by instrumental evidence of neuroplasticity (EEG analysis) and autonomic nervous system modulation (HRV and IRT). Notably, EEG microstate analysis suggested changes in the functional organization of CNS activity, while IRT and HRV measures indicated ANS activity modulation toward parasympathetic dominance. Although preliminary and limited to a single patient, these findings support the potential of EMS as a non-invasive, home-based tool for neuromodulation in the rehabilitation of MS patients. Further research with larger cohorts is needed to confirm these results, evaluate the long-term sustainability of the effects, and optimize stimulation parameters. The integration of wearable technologies such as EMS into clinical practice may represent an innovative opportunity to personalize rehabilitative interventions for neurological patients.

## Figures and Tables

**Table 1:** Targeted muscle groups and stimulation parameters (pulse width) used in the EMS for the patient. Each muscle’s stimulation was adjusted based on individual characteristics. (µs = microseconds).

Muscle/Region	Movement	Pulse Width (µs)
Right abdominals	flexion	50
Left abdominals	flexion	50
Right hip	extension	25
Right hip	flexion	65
Right hip	external rotation	80
Left hip	extension	25
Left hip	flexion	65
Left hip	external rotation	95
Right ankle	dorsiflexion	95
Right ankle	plantar flexion	25
Left ankle	dorsiflexion	110
Left ankle	plantar flexion	25
Right lumbar spine	extension	25
Left lumbar spine	extension	25
Right thoracic spine	extension	60
Left thoracic spine	extension	60
Upper right thoracic spine	extension	35
Upper left thoracic spine	extension	35
Right knee	extension	25
Right knee	flexion	65
Left knee	extension	25
Left knee	flexion	75
Right elbow	extension	50
Right elbow	flexion	25
Left elbow	extension	60
Left elbow	flexion	25
Right wrist	dorsiflexion	65
Left wrist	dorsiflexion	80
Right shoulder	abduction	35
Left shoulder	abduction	35

**Table 2:** Clinical scales evaluated for the patient.

Test	T0	T1
STS	8 s	6 s
TUG	59 s	59 s
Right Lower Limb Clonus Test	inexhaustible	absent
Left Lower Limb Clonus Test	inexhaustible	exhaustible
Right Leg Pendulum Test	5	3
Left Leg Pendulum Test	25	17
VAS	5/10	5/10
MAS for Right Lower Limb Tone Test	3/4	2/4
MAS for Left Lower Limb Tone Test	2/4	1/4
Penn Spasm Frequency Scale	3/4	2/4
Modified Fatigue Impact Test Scales (MFIS)	43/84	40/84

**Table 3:** EEG power bands evaluated during the different experimental phases.

EEG Metrics	Channels	Pre Stim T0	Post Stim T0	Pre Stim T1	Post Stim T1
θ-band	1	0.6297	0.2471	0.1541	0.4904
	2	0.0502	0.0455	0.1162	0.3359
	3	0.6395	0.2243	0.1366	0.4398
	4	0.0118	0.0948	0.9916	0.2592
	5	0.6245	0.2591	0.1447	0.4699
	6	0.8454	0.2299	0.1237	0.2261
	7	0.3398	0.2461	0.1744	0.4768
	8	0.0129	0.1568	0.1801	0.3281
α-band	1	0.0238	0.1154	0.1068	0.2887
	2	0.0317	0.1197	0.8221	0.1647
	3	0.0428	0.1079	0.8621	0.2732
	4	0.692	0.0735	0.5698	0.1495
	5	0.0418	0.1026	0.8862	0.2736
	6	0.524	0.0029	0.6416	0.1236
	7	0.0269	0.0034	0.9360	0.1429
	8	0.594	0.0827	0.7530	0.0036
β-band	1	0.2629	0.7435	0.8660	1.5326
	2	0.0175	0.1096	0.6640	0.1143
	3	0.2654	0.8362	0.6864	1.4620
	4	0.5418	0.2572	0.4485	0.0965
	5	0.2372	0.0822	0.7101	1.4632
	6	0.4895	0.7814	0.5019	0.1043
	7	0.1168	0.7554	0.7129	1.4419
	8	0.4774	0.0543	0.5290	0.1283
δ-band	1	0.3107	0.51866	0.1173	0.4839
	2	0.3828	0.1702	0.0990	0.0165
	3	0.2680	0.4987	0.0858	0.5200
	4	0.2217	0.2305	0.0650	0.1330
	5	0.3213	1.1299	0.1007	0.5440
	6	0.0094	0.7936	0.0890	0.0191
	7	0.0269	0.5106	0.0940	0.5260
	8	0.4633	0.7571	0.1018	0.0261
γ-band	1	0.0118	0.3527	0.1173	0.5839
	2	0.0123	0.1077	0.0990	0.0165
	3	0.0105	0.3497	0.0858	0.5200
	4	0.0103	0.2079	0.0650	0.0133
	5	0.0105	0.0216	0.1007	0.5440
	6	0.0102	0.1082	0.0890	0.0191
	7	0.0004	0.3403	0.0940	0.5260
	8	0.0005	0.0142	0.1018	0.0261

**Table 4:** EEG microstates metrics evaluated during the different experimental phases.

Microstates Metrics	Pre Stim T0	Post Stim T0	Pre Stim T1	Post Stim T1
Mean_Dur	0.3381	0.0516	0.1266	0.0524
GFP	19.042	14.355	18.883	27.714
Hurst	0.7656	0.8707	0.7180	0.6906
GEV	0.8855	0.6604	0.9003	0.8203
Coverage state 1	0.5160	0.9313	0.2397	0.1942
Coverage state 2	0.0289	0.0139	0.1601	0.2308
Coverage state 3	0.4248	0.0373	0.5742	0.2408
Coverage state 4	0.0303	0.0176	0.0260	0.3342
Occurrence state 1	1.1312	0.6416	2.6717	3.7819
Occurrence state 2	0.5913	0.3418	1.8557	4.8952
Occurrence state 3	0.5193	0.6507	2.8234	5.1026
Occurrence state 4	0.7122	0.3649	0.5299	5.2943

**Table 5:** HRV metrics evaluated during the different experimental phases. The arrows in the table represent the trend of the metrics during the experimental sessions (i.e., ↑ indicates the increasing, whereas ↓ the decreasing).

ECG Metrics	Pre Stim	Post Stim T0	Pre Stim T1	Post Stim T1	Trend
SDNN	0.1557	0.1313	0.0692	0.0616	↓
RMSSD	0.1884	0.2023	0.0837	0.0648	↓
LF	33.20	15.1785	23.5221	5.7115	↓
HF	10243	11895	4517	263.3741	↓
LF/HF	0.0032	0.0128	0.0052	0.0217	↑

**Table 6:** IRT metrics evaluated during the different experimental phases. The arrows in the table represent the trend of the metrics during the experimental sessions (i.e., ↑ indicates the increasing, whereas ↓ the decreasing).

ROIs	IRT Metrics	Post Stim T0	Post Stim T1	Post Stim T1-Pre Stim (%)	Trend
Nose Tip	T_avg	36.663	31.535	−13.987	↓
	T_std	0.170	0.504	196.471	↑
	T_kurt	2.414	2.995	24.068	↑
	T_skew	0.166	0.645	288.554	↑
	T_SampEn	0.090	0.239	165.556	↑
	T_LF	183.124	53.353	−70.865	↓
	T_HF	15.589	4.229	−72.872	↓
	T_LF/HF	11.747	12.614	7.381	↑
Right Nostril	T_avg	36.138	31.976	−11.517	↓
	T_std	0.304	0.636	109.211	↑
	T_kurt	1.734	2.365	36.390	↑
	T_skew	0.238	0.253	6.303	↑
	T_SampEn	0.264	0.190	−28.030	↓
	T_LF	177.346	56.098	−68.368	↓
	T_HF	15.081	4.473	−70.340	↓
	T_LF/HF	11.759	12.541	6.650	↑
Left Nostril	T_avg	36.334	34.245	−5.749	↓
	T_std	0.170	0.627	268.824	↑
	T_kurt	2.679	2.359	−11.945	↓
	T_skew	0.185	0.208	12.432	↑
	T_SampEn	0.433	0.126	−70.901	↓
	T_LF	180.897	63.624	−64.829	↓
	T_HF	15.327	5.033	−67.163	↓
	T_LF/HF	11.802	12.643	7.126	↑
Chin	T_avg	36.043	35.633	−1.138	↓
	T_std	0.099	0.312	215.152	↑
	T_kurt	2.595	2.320	−10.597	↓
	T_skew	−0.541	−0.057	−89.464	↓
	T_SampEn	0.167	0.118	−29.341	↓
	T_LF	174.636	66.261	−62.058	↓
	T_HF	14.497	5.293	−63.489	↓
	T_LF/HF	11.683	12.518	7.147	↑
Perioral	T_avg	36.692	35.142	−4.224	↓
	T_std	0.105	0.378	260.000	↑
	T_kurt	2.177	2.697	23.886	↑
	T_skew	0.355	0.225	−36.620	↓
	T_SampEn	0.341	0.058	−82.991	↓
	T_LF	11.728	12.641	7.785	↑
	T_HF	15.610	5.229	−66.502	↓
	T_LF/HF	183.082	66.093	−63.900	↓
Corrugator	T_avg	36.02	34.69	−3.692	↓
	T_std	0.161	0.325	101.863	↑
	T_kurt	2.061	2.909	41.145	↑
	T_skew	−0.464	0.225	−148.491	↓
	T_SampEn	0.016	0.006	−62.500	↓
	T_LF	174.750	63.743	−63.523	↓
	T_HF	14.968	5.058	−66.208	↓
	T_LF/HF	11.675	12.603	7.949	↑

## Data Availability

The data are available upon request to the corresponding author.

## Abbreviations

The following abbreviations are used in this manuscript:

MS	Multiple sclerosis
ANS	Autonomic Nervous System
CNS	Central Nervous System
EMS	Exopulse Mollii suit
EEG	Electroencephalography
HRV	Heart Rate Variability
IRT	Infrared Thermography
TMS	Transcranial Magnetic Stimulation
VNS	Vagus Nerve Stimulation
TENS	Transcutaneous Electrical Nerve Stimulation
ICF	International Classification of Functioning
MAS	Modified Ashworth Scale
SNS	Sympathetic Nervous System
PNS	Parasympathetic Nervous System
EDSS	Expanded Disability Status Scale
MRC	Medical Research Council
STS	Sit to Stand
TUG	Timed Up and Go
VAS	Visual Analog Scale
MFIS	Modified Fatigue Impact Test Scales
ICA	Independent Component Analysis
GFP	Global Field Power
GEV	Global Explained Variance
SDNN	Standard Deviation of Normal Heartbeat Intervals
RMSSD	Root Mean Square of Successive Differences
LF	Low-Frequency Power
HF	High-Frequency Power
LF/HF	Low-Frequency to High-Frequency Ratio
ROI	Regions of Interest
T_avg	Mean Temperature
T_std	Standard Deviation Temperature
T_kurt	Kurtosis Temperature
T_skew	Skewness Temperature
T_SampEn	Sample Entropy Temperature
T_LF	Low-Frequency Power Temperature
T_HF	High-Frequency Power Temperature
T_LF/HF	LF/HF Ratio Temperature

## References

[1] Boudarham J., Hameau S., Zory R., Hardy A., Bensmaïl D., Roche N. (2016). Coactivation of Lower Limb Muscles During Gait in Patients with Multiple Sclerosis. PLoS ONE.

[2] Brandão P.d.M.F., Lino T.B., Oliveira R.T.d., Parra A.V., Andrade P.H.M., Christofoletti G. (2022). Age, Motor Dysfunction and Neuropsychiatric Symptoms Impact Quality of Life in Multiple Sclerosis. Rev. Bras. Enferm..

[3] Oh J., Vidal-Jordana Á., Montalbán X. (2018). Multiple Sclerosis: Clinical Aspects. Curr. Opin. Neurol..

[4] Lambercy O., Fluet M.-C., Lamers I., Kerkhofs L., Feys P., Gassert R. Assessment of Upper Limb Motor Function in Patients with Multiple Sclerosis Using the Virtual Peg Insertion Test: A Pilot Study. Proceedings of the 2013 IEEE 13th International Conference on Rehabilitation Robotics (ICORR).

[5] LaRocca N.G. (2011). Impact of Walking Impairment in Multiple Sclerosis. Patient Patient-Centered Outcomes Res..

[6] Wiendl H., Gold R., Berger T., Derfuss T., Linker R.A., Mäurer M., Aktaş O., Baum K., Berghoff M., Bittner S. (2021). Multiple Sclerosis Therapy Consensus Group (MSTCG): Position Statement on Disease-Modifying Therapies for Multiple Sclerosis (White Paper). Ther. Adv. Neurol. Disord..

[7] Centonze D., Leocani L., Feys P. (2020). Advances in Physical Rehabilitation of Multiple Sclerosis. Curr. Opin. Neurol..

[8] Chaves A.R., Devasahayam A.J., Riemenschneider M., Pretty R.W., Ploughman M. (2020). Walking Training Enhances Corticospinal Excitability in Progressive Multiple Sclerosis—A Pilot Study. Front. Neurol..

[9] Swanson C.W. (2023). Links Between Neuroanatomy and Neurophysiology with Turning Performance in People with Multiple Sclerosis. Sensors.

[10] Huang R. (2024). Paired Vagus Nerve Stimulation Drives Precise Remyelination and Motor Recovery After Myelin Loss. bioRxiv.

[11] Ryan C., King R., Robinson V., Punt D., Dinse H.R., Grunenberg C., Johnson M., Martin D. (2016). Transcutaneous Electrical Nerve Stimulation Using an LTP-like Repetitive Stimulation Protocol for Patients with Upper Limb Complex Regional Pain Syndrome: A Feasibility Study. Hand Ther..

[12] Rosenbaum P., Stewart D. (2004). The World Health Organization International Classification of Functioning, Disability, and Health: A Model to Guide Clinical Thinking, Practice and Research in the Field of Cerebral Palsy. Proceedings of the Seminars in Pediatric Neurology.

[13] Stein C., Fritsch C.G., Robinson C., Sbruzzi G., Plentz R.D.M. (2015). Effects of Electrical Stimulation in Spastic Muscles After Stroke: Systematic Review and Meta-Analysis of Randomized Controlled Trials. Stroke.

[14] Schuhfried O., Crevenna R., Fialka-Moser V., Paternostro-Sluga T. (2012). Non-Invasive Neuromuscular Electrical Stimulation in Patients with Central Nervous System Lesions: An Educational Review. J. Rehabil. Med..

[15] Mahmood A., Veluswamy S.K., Hombali A., Mullick A., Manikandan N., Solomon J.M. (2019). Effect of Transcutaneous Electrical Nerve Stimulation on Spasticity in Adults with Stroke: A Systematic Review and Meta-Analysis. Arch. Phys. Med. Rehabil..

[16] Fu X., Wang Y., Wang C., Wu H., Li J., Li M., Ma Q., Yang W. (2018). A Mixed Treatment Comparison on Efficacy and Safety of Treatments for Spasticity Caused by Multiple Sclerosis: A Systematic Review and Network Meta-Analysis. Clin. Rehabil..

[17] Perpetuini D., Russo E.F., Cardone D., Palmieri R., De Giacomo A., Pellegrino R., Merla A., Calabrò R.S., Filoni S. (2023). Use and Effectiveness of Electrosuit in Neurological Disorders: A Systematic Review with Clinical Implications. Bioengineering.

[18] Wong C., Torabi T.P., Mortensen K., Michelsen J. (2018). The Mollii-Suit^®^—A Novel Method Using Reciprocal Inhibition in Children with Cerebral Palsy, Gross Motor Function Classification System IV-V: A 6-Month Prospective Study. Toxicon.

[19] Wagner J., Makeig S., Hoopes D.J., Gola M. (2019). Can Oscillatory Alpha-Gamma Phase-Amplitude Coupling Be Used to Understand and Enhance TMS Effects?. Front. Hum. Neurosci..

[20] Schaworonkow N., Triesch J., Ziemann U., Zrenner C. (2019). EEG-Triggered TMS Reveals Stronger Brain State-Dependent Modulation of Motor Evoked Potentials at Weaker Stimulation Intensities. Brain Stimul..

[21] Bréchet L., Michel C.M. (2022). EEG Microstates in Altered States of Consciousness. Front. Psychol..

[22] Wang T., Chen Y.-H. (2023). Exploring the Role of Visual Guidance in Motor Imagery-Based Brain-Computer Interface: An EEG Microstate-Specific Functional Connectivity Study. Bioengineering.

[23] Rajkumar R., Farrher E., Mauler J., Sripad P., Brambilla C.R., Kops E.R., Scheins J., Dammers J., Lerche C., Langen K. (2018). Comparison of EEG Microstates with Resting State fMRI and FDG-PET Measures in the Default Mode Network via Simultaneously Recorded Trimodal (PET/MR/EEG) Data. Hum. Brain Mapp..

[24] Cruz J.R.d., Favrod O., Roinishvili M., Chkonia E., Brand A., Möhr C., Figueiredo P., Herzog M.H. (2020). EEG Microstates Are a Candidate Endophenotype for Schizophrenia. Nat. Commun..

[25] Bréchet L., Brunet D., Birot G., Gruetter R., Michel C.M., Jorge J. (2019). Capturing the Spatiotemporal Dynamics of Self-Generated, Task-Initiated Thoughts with EEG and fMRI. Neuroimage.

[26] Wang J., Xu L., Ge Q., Xue L., Liu Y., Wu Y., Liu Y., Chen L., Zhuang Y., Geng X. (2023). EEG Microstate Changes During Hyperbaric Oxygen Therapy in Patients with Chronic Disorders of Consciousness. Front. Neurosci..

[27] Lamoš M., Moravkova I., Ondráček D., Bočková M., Rektorová I. (2021). Altered Spatiotemporal Dynamics of the Resting Brain in Mild Cognitive Impairment with Lewy Bodies. Mov. Disord..

[28] Perpetuini D., Croce P., Chiarelli A.M., Cardone D., Zappasodi F., Merla A., Costin H.-N., Magjarević R., Petroiu G.G. (2024). Modifications of Microstates in Resting-State EEG Associated to Cognitive Decline in Early Alzheimer’s Disease Assessed by a Machine Learning Approach. Advances in Digital Health and Medical Bioengineering, Proceedings of the EHB 2023, Bucharest, Romania, 9–10 November 2023.

[29] Reeves J.L., Graff-Radford S.B., Shipman D. (2004). The Effects of Transcutaneous Electrical Nerve Stimulation on Experimental Pain and Sympathetic Nervous System Response. Pain Med..

[30] Binny J., Joshua Wong N.L., Garga S., Lin C.-W.C., Maher C.G., McLachlan A.J., Traeger A.C., Machado G.C., Shaheed C.A. (2019). Transcutaneous Electric Nerve Stimulation (TENS) for Acute Low Back Pain: Systematic Review. Scand. J. Pain.

[31] Shah A.J., Su S., Veledar E., Bremner J.D., Goldstein F.C., Lampert R., Goldberg J., Vaccarino V. (2011). Is Heart Rate Variability Related to Memory Performance in Middle-Aged Men?. Psychosom. Med..

[32] Kleen M., Reitsma B. (2011). Appliance of Heart Rate Variability Biofeedback in Acceptance and Commitment Therapy: A Pilot Study. J. Neurother..

[33] Thomas B.L., Claassen N., Becker P., Viljoen M. (2019). Validity of Commonly Used Heart Rate Variability Markers of Autonomic Nervous System Function. Neuropsychobiology.

[34] Di Credico A., Perpetuini D., Izzicupo P., Gaggi G., Mammarella N., Di Domenico A., Palumbo R., La Malva P., Cardone D., Merla A. (2024). Predicting Sleep Quality through Biofeedback: A Machine Learning Approach Using Heart Rate Variability and Skin Temperature. Clocks Sleep.

[35] Lin W.-H., Wu D., Li C., Zhang H., Zhang Y.-T., Zhang Y.-T. (2014). Comparison of Heart Rate Variability from PPG with That from ECG. Proceedings of the International Conference on Health Informatics.

[36] Iarlori S., Perpetuini D., Tritto M., Cardone D., Tiberio A., Chinthakindi M., Filippini C., Cavanini L., Freddi A., Ferracuti F. (2024). An Overview of Approaches and Methods for the Cognitive Workload Estimation in Human–Machine Interaction Scenarios through Wearables Sensors. BioMedInformatics.

[37] Fricová J., Janatová M., Albrecht J., Mareš T., Rokyta R., Masopust V., Anders M. (2021). A Prospective Single-Center Study of the Effects of Repetitive Transcranial Magnetic Stimulation at 2-Week Follow-Up in 17 Patients with Chronic Orofacial Pain Diagnosed by Infrared Thermography. Med. Sci. Monit..

[38] Neves E.B., Vilaça-Alves J., Rosa C., Reis V.M. (2015). Thermography in Neurologic Practice. Open Neurol. J..

[39] Znamenskaya I.A., Koroteeva E.Y., Isaychev A., Chernorizov A.M. Thermography-Based Remote Detection of Psycho-Emotional States. Proceedings of the 14th Quantitative InfraRed Thermography Conference.

[40] Mouazen B., Bendaouia A., Abdelwahed E.H., De Marco G. (2025). Machine learning and clinical EEG data for multiple sclerosis: A systematic review. Artif. Intell. Med..

[41] Adamec I., Habek M. (2013). Autonomic Dysfunction in Multiple Sclerosis. Clin. Neurol. Neurosurg..

[42] Ring E.F.J., Ammer K. (2012). Infrared Thermal Imaging in Medicine. Physiol. Meas..

[43] Diakides M., Bronzino J.D., Peterson D.R. (2012). Medical Infrared Imaging: Principles and Practices.

[44] Marins J.C.B., Formenti D., Costa C.M.A., de Andrade Fernandes A., Sillero-Quintana M. (2015). Circadian and Gender Differences in Skin Temperature in Militaries by Thermography. Infrared Phys. Technol..

[45] Croce P., Zappasodi F., Marzetti L., Merla A., Pizzella V., Chiarelli A.M. (2018). Deep Convolutional Neural Networks for Feature-Less Automatic Classification of Independent Components in Multi-Channel Electrophysiological Brain Recordings. IEEE Trans. Biomed. Eng..

[46] Murray M.M., Brunet D., Michel C.M. (2008). Topographic ERP Analyses: A Step-by-Step Tutorial Review. Brain Topogr..

[47] Krzanowski W.J., Lai Y.T. (1988). A Criterion for Determining the Number of Groups in a Data Set Using Sum-of-Squares Clustering. Biometrics.

[48] Pascual-Marqui R.D., Michel C.M., Lehmann D. (1995). Segmentation of Brain Electrical Activity into Microstates: Model Estimation and Validation. IEEE Trans. Biomed. Eng..

[49] Michel C.M., Koenig T. (2018). EEG Microstates as a Tool for Studying the Temporal Dynamics of Whole-Brain Neuronal Networks: A Review. Neuroimage.

[50] Cardone D., Spadolini E., Perpetuini D., Filippini C., Chiarelli A.M., Merla A. (2021). Automated Warping Procedure for Facial Thermal Imaging Based on Features Identification in the Visible Domain. Infrared Phys. Technol..

[51] Alenazy M., Daneshgar Asl S., Petrigna L., Feka K., Alvarez E., Almuklass A.M., Enoka R.M. (2021). Treatment with Electrical Stimulation of Sensory Nerves Improves Motor Function and Disability Status in Persons with Multiple Sclerosis: A Pilot Study. J. Electromyogr. Kinesiol..

[52] Amatya B., Young J., Khan F. (2018). Non-Pharmacological Interventions for Chronic Pain in Multiple Sclerosis. Cochrane Database Syst. Rev..

[53] Wahls T.L., Reese D., Kaplan D., Darling W.G. (2010). Rehabilitation with Neuromuscular Electrical Stimulation Leads to Functional Gains in Ambulation in Patients with Secondary Progressive and Primary Progressive Multiple Sclerosis: A Case Series Report. J. Altern. Complement. Med..

[54] Miller L., Mattison P., Paul L., Wood L. (2007). The Effects of Transcutaneous Electrical Nerve Stimulation (TENS) on Spasticity in Multiple Sclerosis. Mult. Scler. J..

[55] Schabrun S.M., Ridding M.C., Galea M.P., Hodges P.W., Chipchase L.S. (2012). Primary Sensory and Motor Cortex Excitability Are Co-Modulated in Response to Peripheral Electrical Nerve Stimulation. PLoS ONE.

[56] Khanna A., Pascual-Leone A., Michel C.M., Farzan F. (2015). Microstates in resting-state EEG: Current status and future directions. Neurosci. Biobehav. Rev..

[57] do Amaral Sartori S., Stein C., Coronel C.C., Macagnan F.E., Plentz R.D.M. (2018). Effects of Transcutaneous Electrical Nerve Stimulation in Autonomic Nervous System of Hypertensive Patients: A Randomized Controlled Trial. Curr. Hypertens. Rev..

[58] Stein C., Dal Lago P., Ferreira J.B., Casali K.R., Plentz R.D.M. (2011). Transcutaneous Electrical Nerve Stimulation at Different Frequencies on Heart Rate Variability in Healthy Subjects. Auton. Neurosci..

[59] Ioannou S., Gallese V., Merla A. (2014). Thermal Infrared Imaging in Psychophysiology: Potentialities and Limits. Psychophysiology.

[60] Perpetuini D., Russo E.F., Cardone D., Palmieri R., Filippini C., Tritto M., Pellicano F., De Santis G.P., Pellegrino R., Calabrò R.S. (2022). Psychophysiological Assessment of Children with Cerebral Palsy during Robotic-Assisted Gait Training through Infrared Imaging. Int. J. Environ. Res. Public Health.

